# Women Referred for Liver Transplant Are Less Likely to Be Transplanted Irrespective of Socioeconomic Status

**DOI:** 10.3389/ti.2023.11667

**Published:** 2023-11-02

**Authors:** Emmanouil Giorgakis, Martha M. Estrada, Allison Wells, Mauricio Garcia Saenz de Sicilia, Matthew Deneke, Raj Patel, Gary Barone, Lyle Burdine, Mary K. Rude

**Affiliations:** ^1^ Division of Solid Organ Transplantation, Department of Surgery, University of Arkansas for Medical Sciences, Little Rock, AR, United States; ^2^ Division of Gastroenterology and Hepatology, Department of Medicine, University of Arkansas for Medical Sciences, Little Rock, AR, United States

**Keywords:** disparities, liver transplant, social vulnerability index, sex disparities, public policy

Dear Editors,

Liver transplantation is the standard of care for end-stage liver disease (ESLD) and transplant oncology patients. Given the organ shortage, equitable organ distribution is key. Recent studies have repeatedly reported that, in the US, waitlisted patients of female sex are less likely to be transplanted and more likely to die awaiting a liver transplant [1, 2]. This has been largely attributed to an imperfect model for end-stage liver disease (MELD) scoring systems and donor-recipient size mismatch [1, 3, 4].

After obtaining institutional board review exemption (IRB 275415), we explored socioeconomic and sex-related disparities of patients referred for liver transplant at Arkansas’ single liver transplant institution. The Centers for Disease Control and Prevention (CDC)/Agency for Toxic Substances and Disease Registry (ATCSDR) Social Vulnerability Index (SVI) was employed as surrogate indicator of socioeconomic status [5]. Social vulnerability refers to the resilience of a population when confronted by a health stressor, be it a disease outbreak or a natural or human-caused disaster. CDC/ATSDR SVI database “*can help communities prepare for and recover from public health emergencies, and prevent adverse effects among socially vulnerable populations, such as emotional distress, loss of property, illness, and death*” [5]. The SVI calculation encompasses parameters reflecting a community’s *socioeconomic* (e.g., poverty, unemployment, *per capita* income, education, and health insurance), *population* (e.g., children or elderly, disability, single parent, minority, limited English), and *housing/transportation* (e.g., mobile homes, crowding, no vehicle, living in group quarters) *vulnerability*. Data was sourced from the Arkansas Clinical Data Repository.

Patients with less than 1 year follow-up or missing data were excluded. SVI scores were assigned by patient’s ZIP code, which reflects the patient’s location of residence. The patients were split into SVI quartiles, based on SVI median and interquartile range. Logistic regression was performed for enlisting, adjusted for SVI quartile, age, sex, body mass index, and insurance payor. A Fine-Gray survival model was built, with liver transplant as the primary outcome and death a competing event controlled for sex, SVI quartile, and insurance. Analyses were conducted using R software (4.1.0) and STATA version (17.0).

Study period was from 1st January 2019 to 31st December 2022. The study population included *N* = 779 patients who had been referred to our center during that time for liver transplant evaluation. 43.2% (*N* = 336) of these patients were female. Logistic regression analysis indicated that, irrespective of SVI quartile, male sex and private insurance were independent predictors favoring liver transplantation (odds ratio [OR] 2.73; 95% CI, 1.70–4.52, and 2.2; 95% CI, 1.35–3.70, respectively; Table 1). Likewise, on Fine-Gray analysis adjusted for SVI quartile, male sex and Medicare/Medicaid insurance payor were independent risk factors (OR 2.38; 95% CI, 1.53–3.70, and 0.48; 95% CI, 0.30–0.76, respectively) (Table 2). *Waitlisted* male patients with private insurance were more likely to get transplanted and survive after a liver transplant. What is more, male sex patients *referred* for liver transplant were found more likely to be *evaluated* (OR 1.76, *p* < 0.001), *enlisted* (OR 2.07, *p* < 0.001) and *transplanted* (OR 2.55, *p* < 0.001) compared to their female counterparts (Supplementary Data).

**TABLE 1:** Multivariate analysis of liver transplant outcome.

	Odds ratios (OR)	95% CI	*p*
Male Sex	**2.73**	**1.70–4.52**	**<0.001**
Private Insurance payor	**2.2**	**1.35–3.70**	**0.002**
SVI quartile			
(Intercept)	0.16	0.03–0.78	0.025
2	0.56	0.27–1.12	0.108
3	1.09	0.63–1.92	0.756
4	1.09	0.60–1.99	0.769
Age	0.98	0.96–1.00	0.061

Bold value indicates the male sex and private insurance independently favored liver transplant (odds ratio [OR] 2.73; 95% CI, 1.70–4.52, and 2.2; 95% CI, 1.35–3.70, respectively).

**TABLE 2:** Fine gray competing risk survival analysis of patients referred for liver transplant.

	OR	95% CI	*p*
Medicare/Medicaid	**0.48**	**0.30–0.76**	**0.002**
Male Sex	**2.38**	**1.53–3.70**	**<0.001**
SVI quartile			
2 (0.53–0.75)	0.59	0.30–1.13	0.112
3 (0.76–0.81)	1.04	0.64–1.71	0.864
4 (≥0.81)	1.00	0.59–1.69	0.994

Bold value indicates the male sex favored liver transplantation (OR 2.38; 95% CI, 1.53–3.70). Medicare/Medicaid insurance payor decreased the odds getting a liver transplant (OR 0.48; 95% CI, 0.30–0.76).

In conclusion, our study indicates that, in the population and period studied, there are sex related barriers in the liver transplant process. These obstacles may prevent female sex patients from entering and completing liver transplant evaluation. This gap may be ascribed to *functional status assessment* barriers [2], e.g., higher perceived frailty among females, particularly elderly; *clinical*, e.g., higher female prevalence of nonalcoholic steatohepatitis (NASH), with NASH known to be associated with higher surgical risk; *social* [1, 2], e.g., work or family obligations preventing completion of the evaluation process; the *stigma* of alcohol excess [1, 2]; or *geographic*, i.e., within minority groups residing in remote locations. Beyond introducing remedies such as scoring system upgrades accounting for patient’s sex [1, 2], it is also necessary to address sex-based barriers presenting early on in the liver transplant referral and evaluation process [2]. A good start may be the 1) creation of national or regional liver disease/ESLD registries in order to achieve better data granularity; 2) introduction of transplant referral and evaluation efficiency metrics (e.g., time from referral to decision over enlisting) [2]; 3) implementation of objective frailty testing methods [2]; and 4) provisions for a more flexible evaluation process, tailored to individual socioeconomic, geographic, and cultural needs.

Limitations of this pilot study were its limited sample, retrospective nature, and the inclusion of liver transplant referrals to a single US transplant institution.

## Data Availability

The raw data supporting the conclusion of this article will be made available by the authors, without undue reservation.

## Abbreviations

ATCDR, agency for toxic substances and disease registry; CDC, centers for disease control and prevention; ESLD, end-stage liver disease; MELD, model for end-stage liver disease; NASH, non-alcoholic steatohepatitis; SVI, social vulnerability index.
