# Supplementary material for: Women Referred for Liver Transplant Are Less Likely to Be Transplanted Irrespective of Socioeconomic Status
Source: Transpl Int. 2023 Nov 2;36:11667. doi: 10.3389/ti.2023.11667 (PMC10651715; doi:10.3389/ti.2023.11667)
Supplement: Supplementary file 1 [file Table1.DOCX]

**Table 1.** Demographics per Sex of Patients Referred for Liver Transplant

|  | **Total (%)** | **Female (%)** | **Male (%)** | **OR** | **p** |
| --- | --- | --- | --- | --- | --- |
| N | **779** | **336 (43.1)** | **443 (56.9)** |  |  |
| **Age (at time of referral)**  median [IQR] |  | 57  [48.00, 64.00] | 57  [50.00, 64.00] | 1.01 | 0.261 |
| **Evaluated** | **412 (52.9)** | **151 (44.9)** | **261 (58.9)** | **1.76** | **<0.001** |
| **Waitlisted** | **143 (18.4)** | **42 (12.5)** | **101 (22.8)** | **2.07** | **<0.001** |
| **Transplanted** |  | **26 (7.7)** | **78 (17.6)** | **2.55** | **<0.001** |
| **Referral Status** |  |  |  |  |  |
| ***Eligible*** |  | **31 (9.2)** | **81 (18.3)** | **Ref.** | **0.001** |
| *Ineligible* |  | 292 (86.9) | 340 (76.7) | 0.45 [0.28;0.69] |  |
| *Removed* |  | 13 (3.9) | 22 (5.0) | 0.65 [0.29;1.48] |  |
| **Overall deaths** | 194 (100) | 92 (27.4) | 102 (23.0) | 0.79 [0.57;1.10] | 0.942 |
| *Post-LT* | 6 (3.09) | 2 (0.6) | 4 (0.9) | 1.47 [0.27;11.9] | 0.191 |
| *On waitlist* | 16 (8.24) | 7 (2.1) | 9 (2.0) | 0.97 [0.35;2.79] | 1 |
| *Awaiting evaluation* | 80 (41.23) | 41 (12.2) | 39 (8.8) | 0.70 [0.44;1.11] | 0.153 |
| *Declined for LT* | 92 (47.4) | 42 (12.5) | 50 (11.3) | 0.89 [0.57;1.38] | 0.684 |

OR, odds ratio; IQR, interquartile range; LT; liver transplant
